# An Evidence-Based Intervention to Increase *Trypanosoma cruzi*, a Neglected Parasitic Infection, Diagnosis in Rural and Moderate-Size-City US Clinics

**DOI:** 10.1093/ofid/ofaf467

**Published:** 2025-08-14

**Authors:** M K Lynn, Hunter M Boehme, Jeffrey Hall, Patrick Kent, Alain H Litwin, Quang H Pham, Melissa S Nolan, Prisma Chagas Team, Chloe M Rodriguez Ramos, Mary Parker, Hanna Waltz, Kia Zellars, Madeleine M Meyer, Kylie Whittle

**Affiliations:** Department of Epidemiology and Biostatistics, Arnold School of Public Health, University of South Carolina, Columbia, South Carolina, USA; Institute for Infectious Disease Translational Research, University of South Carolina, Columbia, South Carolina, USA; Department of Criminology and Criminal Justice, University of South Carolina, Columbia, South Carolina, USA; School of Medicine, University of South Carolina, Columbia, South Carolina, USA; Department of Family and Preventive Medicine, Prisma Health–Midlands, Columbia, South Carolina, USA; School of Medicine–Greenville, University of South Carolina, Greenville, South Carolina, USA; Department of Medicine, Prisma Health–Upstate, Greenville, South Carolina, USA; School of Medicine–Greenville, University of South Carolina, Greenville, South Carolina, USA; Department of Medicine, Prisma Health–Upstate, Greenville, South Carolina, USA; Department of Family Medicine, Prisma Health--Upstate, Greenville, South Carolina, USA; Department of Epidemiology and Biostatistics, Arnold School of Public Health, University of South Carolina, Columbia, South Carolina, USA; Institute for Infectious Disease Translational Research, University of South Carolina, Columbia, South Carolina, USA

**Keywords:** Chagas disease, *Trypanosoma cruzi*, neglected infectious disease, healthcare access disparity, Latin American health

## Abstract

**Background:**

Chagas disease is a chronic, insidious parasitic infection (*Trypanosoma cruzi*) that slowly develops to irreversible organomegaly over several decades. The disease is traditionally acquired in endemic Latin American countries during childhood; <1% of foreign-born adult residents in the United States have been diagnosed or treated with this potentially fatal disease. Low physician knowledge is a primary factor leading to misdiagnosis.

**Methods:**

Starting in April 2022, a 4-part *T cruzi* clinical education intervention began, which included (*i*) 2 grand rounds presentations to >100 internal medicine providers; (*ii*) implementation of a “clinical Chagas champions program” incorporating 14 key clinical staff at varying departments and administrative levels educated on their specific role related to *T cruzi* screening, diagnosis confirmation, clinical management, and medical billing; (*iii*) connecting clinicians with external, experienced providers to provide guidance during the medically challenging treatment process; and (*iv*) *T cruzi* patient screening at Prisma Health hospitals, family medicine clinics, or affiliated free health clinics. The program's long-term impact was evaluated using a panel Poisson time series statistical model of ordered tests pre- and post-intervention.

**Results:**

For the healthcare system screening initiative, 71 participants were enrolled from across Prisma Health's 21-county region, with a 2.9% Chagas disease seroprevalence detected. Time series analysis of *T cruzi* testing orders within the healthcare system demonstrated a statistically significant increase in ordered tests across the 30 months post-intervention compared to the 51 months prior.

**Conclusions:**

This intervention substantiates the need to pair academic-health partnerships and clinical awareness campaigns to sustainably support long-term *T cruzi* screening in nontraditional areas.

## Teaser

This manuscript describes a multicomponent intervention that sustainably increased clinical diagnosis and treatment for an insidious, neglected parasitic infection. Comprehensive interventions can create a paradigm shift in how providers in rural or moderate-size metropolitan areas approach clinical management for rare pathogens.

## Graphical Abstract

**Graphical Abstract:**
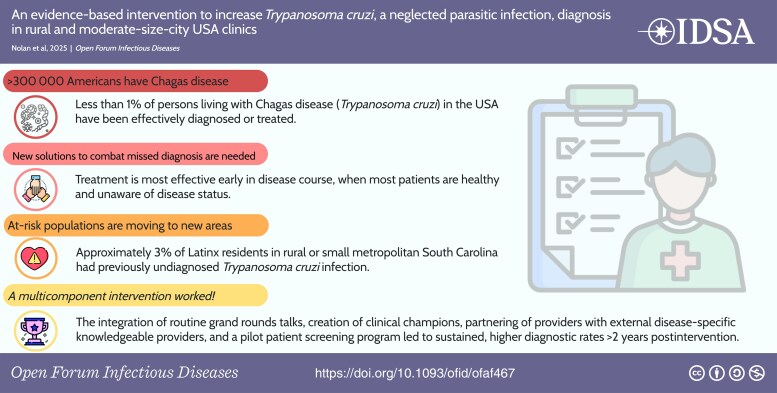
This graphical abstract is also available at Tidbit: https://tidbitapp.io/tidbits/an-evidence-based-intervention-to-increase-trypanosoma-cruzi-a-neglected-parasitic-infection-diagnosis-in-rural-and-moderate-size-city-usa-clinics

The demographics and geography of Latin American persons in the United States (US) has changed considerably in the last decade. The US Census Bureau and Pew Research Center note that the Latin American population increased to 62.1 million in the past decade, a 23% growth compared to 7% overall national population growth, and more than half are now living in areas not traditionally considered Latin American–dominant cities [1, 2]. The Southern US, in particular, has experienced the largest Latinx population growth compared to any other national region (26% increase from 2010 to 2019) [3]. Latin American patients are a vulnerable medical population, afflicted with higher poverty rates, higher unemployment rates, higher uninsured rates, and subsequently higher disease rates nationally (eg, obesity, poor maternal-fetal outcomes, diabetes, stroke) compared to the non-Hispanic White population [4]. Language barriers, lack of healthcare access, and other built environment factors create unique challenges when designing patient population–focused clinical screening or intervention programs. Academic health partnerships are a growing initiative that facilitate cooperation between clinical practice and academic disciplines to improve the overall care of vulnerable communities, and emerging evidence has demonstrated their utility in enhancing sustainable public health programs geared toward the Latin American population [5–9].

Caused by the parasitic protozoan *Trypanosoma cruzi*, Chagas disease is a largely asymptomatic vector-borne infection typically acquired in childhood that, if untreated, insidiously leads to dilated cardiomyopathy or organomegaly in a third of cases. Transmitted by insect vectors endemic throughout the Americas, >6 million persons globally are living with this infection; however, less than 1% have received potentially lifesaving treatment [10, 11]. This slowly progressing subclinical disease develops over decades, affording healthcare providers the opportunity to diagnose infection early before it causes irreversible cardiac or organ damage [12]. Sadly, treatment is not effective once advanced disease stages manifest, making early identification in otherwise healthy adults a public health priority [12].

Chagas disease is a neglected public health concern in the US, with an estimated 288 000 human cases, and 57 000 cases of dilated Chagas cardiomyopathy [13]. Nationally, <1% of cases have been diagnosed and <0.3% have been treated [14]. Due to lack of early identification and treatment, the US healthcare system accumulates up to 130 million dollars annually in Chagas disease–related healthcare costs [15, 16]. Despite these healthcare and economic impacts, no national surveillance exists. Few community-based serology studies have been performed in the US to estimate local prevalence and identify at-risk epidemiologic profiles. These include programs in Los Angeles, Washington, DC, and Boston, calculating prevalence ranges between 0.97% and 3.8% [15, 17, 18]. These programs have provided evidence of notable case burdens in large metropolitan areas, setting the stage for enhanced provider awareness and Chagas disease patients' continuum of care postdiagnosis [13, 18, 19]. However, none have been performed among moderate metropolitan and/or rural Latin American populations—a growing, vulnerable population in the US.

Medical provider knowledge studies have consistently demonstrated low disease knowledge nationally, with providers in moderate metropolitan to rural areas demonstrating lower levels of *T cruzi* clinical knowledge [20–23]. While *T cruzi* educational talks can temporarily increase provider knowledge [21], their long-term impact or their ability to confer increased diagnosis/treatment of infected patients is unknown. Therefore, this study sought to develop, execute, and evaluate the sustainability of a multicomponent *T cruzi*–focused clinician intervention that aimed to increase the rate of ordered Chagas disease diagnostics. This study was implemented within a southern US healthcare system that predominately serves rural residents, as Latinx resident migration is increasing in rural areas or moderate-size southern US cities [1]. Last, a study subaim was to estimate Chagas disease seroprevalence among foreign-born Latin American persons in a nontraditional, rural setting.

## MATERIALS AND METHODS

This multipart clinical intervention was designed and executed with collaboration between the University of South Carolina and Prisma Health. The University of South Carolina is the state's flagship research university with $309 million in extramural grant funding in 2024 [24]. The University of South Carolina has 100 degree programs in the health sciences spread across 2 medical schools and colleges of public health, nursing school, and social work, whose clinical training is performed at a partnering healthcare system, Prisma Health. Prisma Health is the largest nonprofit healthcare system in South Carolina (SC) and services half the state's population across a 21-county region. The healthcare system comprises 18 hospitals, 2200 physicians, 54 residency and fellowship programs, and 2 750 848 outpatient visits in 2024 [25].

The 4-part clinical intervention began on 1 April 2022 and included (*i*) grand rounds–style provider education; (*ii*) a clinical Chagas champion program; (*iii*) connecting inexperienced providers with experienced external providers; and (*iv*) performing free *T cruzi* screening within the healthcare system to provide hands-on training for local providers. First, 2 hour-long grand rounds presentations to >100 infectious disease and primary care physicians were given on 15 April 2022 and 4 August 2022 (audiences ranged from residents to department chairs); these presentations described the epidemiology, clinical presentation, diagnosis, and treatment of Chagas disease. A clinical Chagas champion program was designed to actively encourage physicians and clinical staff to start testing patients for Chagas disease within the healthcare system. Fourteen key personnel received individualized Chagas disease education as it related to their job-specific role in Chagas disease screening, clinical management, and treatment. These personnel titles included academic vice-chair, clinic director, infectious disease pharmacist, fellow, resident, medical student, clinic manager, research nurse, and medical billing personnel. An example of job-specific duties included having an infectious disease resident develop a “best practices” manual for Chagas disease diagnostic workups. Another example includes working with the clinic manager to help uninsured positive patients enroll in the state's free healthcare program. Third, physicians with *T cruzi*–positive patients were connected with the Centers for Disease Control and Prevention’s (CDC) Division of Parasitic Diseases and Malaria Branch personnel or external physicians in other states with prior experience diagnosing and treating patients with Chagas disease.

To provide hands-on training related to Chagas disease clinical management and provide supportive rationale for prospective routine testing within the healthcare system, a *T cruzi* screening study was executed. Grant funding was acquired to cover associated laboratory tests, cardiac exams, and additional clinical costs for uninsured participants. This screening study was approved by the Prisma Health Institutional Review Board (protocol number 1874166-5). From May to July 2022, Latin American–born persons ≥18 years of age were recruited from 3 distinct clinical settings across 5 metropolitan areas. Metropolitan recruitment sites included the Prisma Health Internal Medicine Clinic West (Greenville, SC) and Good Samaritan Clinics (Columbia, SC; West Columbia, SC). Rural recruitment sites utilized free dental clinics presented by the South Carolina Agricultural Worker Health Program (Lodge, SC; Summerton, SC). Study team members and collaborating physicians explained study details to eligible participants. Interested participants provided written informed consent followed by a small blood sample. Enrolled participants further completed a health survey of demographics, comorbidities, and factors of lifetime Chagas disease exposure risk. Serum samples were tested at the University of South Carolina's Institute for Infectious Disease Translational Research via Wiener Chagatest version 3.0 enzyme-linked immunosorbent assay (ELISA) (Wiener Lab, Rosario, Argentina) and Chagas Stat-Pak (Chembio, Medford, New York) immunochromatographic rapid test. All participants with at least 1 preliminary positive test (either Chagas Stat-Pak or Wiener Chagatest) underwent further confirmatory testing managed by Prisma Health clinicians. Prisma Health confirmatory testing was outsourced to a Clinical Laboratory Improvement Amendments–certified laboratory (ARUP Laboratories, Salt Lake City, Utah) where Hemagen Chagas' Kit ELISA (Hemagen Diagnostics, Columbia, Maryland) was performed. All participants were informed of their results. For all preliminary or confirmed positive participants, the study team facilitated confirmatory testing and facilitated physician follow-up for treatment discussion.

The intervention's effect was statistically appraised using a panel generalized estimating equation (GEE) interrupted time series Poisson model, utilizing a fifth-order autoregressive component to account for serial autocorrelation [26]. Additional interrupted time series count models were executed to provide additional rigor to the analytic plan, as findings from interrupted time series may be sensitive to model specifications [27]. In addition to the GEE model, the following models were computed: a pooled interrupted time series model, panel Poisson interrupted time series model, and panel negative binomial interrupted time series model. Last, counts were collapsed into a binary variable outcome for a panel logistic regression model to assess if there were changes in the chance that a test was ordered during pre- and post-intervention weeks [28]. The cumulative count of *T cruzi* diagnostic tests ordered within the Prisma Health system were noted by week; 7 diagnostic test types/commercial laboratory were included in this analysis. The preintervention period was defined as 1 January 2018 to 31 March 2022; the postintervention period was defined as 1 April 2022 to 31 December 2024. A linear and quadratic trend variable were included to assess confounders that may be associated with time and nonlinear trends, as well as a month variable (January as the referent month) to control for seasonality. The diagnostic tests dictated using a fifth-order lag to control for serial autocorrelation.

## RESULTS

The clinical intervention's first 3 components were executed as detailed in the Materials and Methods. Here we describe the results of the *T cruzi* screening component and the intervention's time series evaluation.

In total, 71 Latin American–born South Carolina residents were recruited and agreed to participate. Recruitment included 76% enrolled in moderate-sized metropolitan health clinics and the remaining 24% from rural, free dental clinic sites. The screening program ended early, due to the unexpected high seroprevalence rate and desire to ensure adequate grant funding for clinical management of positive patients without healthcare insurance. Participant sex was comparable between males (45%) and females (55%), and the median age was 43 years (Table 1). Most participants originated from Mexico and Central America, with the majority of Central American countries represented (Honduras, Guatemala, Nicaragua, El Salvador, Nicaragua, Costa Rica). More than half of individuals had less than secondary school education, and almost all were self-pay (ie, uninsured) (Table 1). While the average length of US residency was 13 years, the level of English proficiency varied among participants.

**Table 1.** Seroprevalence of Chagas Disease and Characteristics of Latin American–Born Study Participants^a^

Characteristic	All Participants (n = 69)	*T cruzi–*Negative Participants (n = 67)	*T cruzi–*Positive Participants (n = 2)^a^
Country/region of origin			
Mexico	40 (58)	39 (58.2)	1 (50)
Central America	19 (27.5)	18 (26.9)	1 (50)
South America	9 (13)	9 (13.4)	0 (0)
Latin American Caribbean	1 (1.4)	1 (1.5)	0 (0)
Age group, y			
18–29	10 (14.5)	10 (14.9)	0 (0)
30–39	18 (26.1)	17 (25.4)	1 (50)
40–49	28 (40.6)	27 (40.3)	1 (50)
50–59	9 (13.0)	9 (13.4)	0 (0)
≥60	4 (5.8)	4 (6)	0 (0)
Education			
Secondary school or less	40 (58)	38 (56.7)	2 (100)
More than secondary school	29 (42)	29 (43.3)	0 (0)
No. of persons living in the household			
1–3	24 (34.8)	22 (32.8)	2 (100)
4–6	32 (46.4)	32 (47.8)	0 (0)
7–9	12 (17.4)	12 (17.9)	0 (0)
Health insurance			
Yes (private/employer, Medicaid)	6 (8.7)	6 (9)	0 (0)
No	63 (91.3)	61 (91)	2 (100)
Reasons for difficulty seeking care			
Language, translation barrier	11 (15.9)	11 (16.4)	0 (0)
Money	3 (4.3)	3 (4.5)	0 (0)
Transportation	7 (10.1)	7 (10.4)	0 (0)
Other	2 (2.9)	3 (4.5)	0 (0)
None	62 (89.9)	45 (67.2)	2 (100)

Data are reported as No. (%) of participants.

^a^Prisma Health confirmatory testing was outsourced to ARUP Laboratories (Salt Lake City, Utah) for Hemagen Chagas' Kit enzyme-linked immunosorbent assay testing. Only those with at least 1 positive University of South Carolina test (Chagas Stat-Pak or Wiener Chagatest version 3.0) had confirmatory testing through Prisma Health (n = 4). This table excludes the 2 participants who initially screened positive but were lost to follow-up before confirmatory testing was obtained.

Commonly reported comorbidities included diabetes mellitus, hypertension, and kidney disease. Approximately 8.5% reported cardiac-related comorbidities including congestive heart failure, history of myocardial infarction, and tachycardia. Over 35% of those enrolled reported experiencing at least 1 symptom consistent with Chagas disease (range, 1–7 symptoms), shown in Table 2. Chagas disease knowledge among participants was low (24%); however, exposure was common as more than half reported ever seeing the “kissing bug” (Reduviidae) disease vector in their lifetime. Among those with Chagas disease knowledge, 2 participants reported having family members with the disease.

**Table 2.** Knowledge of Chagas Disease, Disease Vector Sightings, and Reported Symptoms Among Foreign-Born Latin American Study Participants^a^

Participant Reponses	All Participants (n = 69)	*T cruzi–*Negative Participants (n = 67)	*T cruzi–*Positive Participants (n = 2)^a^
Reported frequently experiencing symptoms			
Foot inflammation, swelling	13 (18.8)	12 (17.9)	1 (50)
Difficulty breathing at rest	12 (17.4)	12 (17.9)	0 (0)
Trouble climbing 2 flights of stairs	10 (14.5)	9 (13.4)	1 (50)
Heart racing at rest	10 (14.5)	9 (13.4)	1 (50)
Physician-reported heart abnormality	6 (8.7)	6 (9)	0 (0)
Difficulty swallowing	12 (17.4)	12 (17.9)	0 (0)
Constipation >3 d	12 (17.4)	12 (17.9)	0 (0)
Physician-reported enlarged colon	1 (1.4)	1 (1.5)	0 (0)
Reported belief that Chagas can cause serious health issues			
Yes	14 (20.3)	14 (20.9)	0 (0)
No	1 (1.4)	1 (1.5)	0 (0)
Maybe/not sure	6 (8.7)	4 (6)	0 (0)
Reported belief that those with Chagas should take treatment			
Yes	20 (29)	20 (29.9)	0 (0)
No	0 (0)	0 (0)	0 (0)
Maybe/not sure	2 (2.9)	2 (3)	0 (0)
Ever heard of Chagas disease?			
Yes	15 (21.7)	15 (22.4)	0 (0)
No	53 (76.8)	51 (76.1)	2 (100)
Maybe	1 (1.4)	1 (1.5)	0 (0)
Ever seen a kissing bug?			
Yes	36 (52.2)	34 (50.7)	2 (100)
No	27 (39.1)	27 (40.3)	0 (0)
Not sure	4 (5.8)	4 (6)	0 (0)

Data are reported as No. (%) of participants.

^a^This table excludes the 2 participants who initially screened positive but were lost to follow-up before confirmatory testing was obtained.

The *T cruzi* screening study identified 6 persons with at least 1 reactive test result. Of these 6 positive cases, 4 people returned to the clinic for confirmatory testing, and 2 were lost to follow-up (Table 2). Prisma Health–ordered diagnostic tests confirmed 2 individuals positive for infection. Therefore, a conservative seroprevalence of 2.9% (2 confirmed cases out of 69 participants) was calculated.

Confirmed case-patient 1 was a 45-year-old man originating from rural Mexico with >20 years’ US residency. Known risk factors reported by the individual were history of substandard housing and living in a thatched roof home, along with occupational history of outdoor construction work in daylight hours. The individual had no history of blood, tissue, or organ transplant and did not report ever working outside at night. The participant reported no knowledge of Chagas disease, yet correctly identified the insect vector from pictures. He described no history of being bitten by this insect in his lifetime. Pertinent comorbidities included hypertension and diabetes mellitus. Symptoms potentially consistent with Chagas disease included frequent peripheral edema, dyspnea, heart palpitations at rest, and difficulty climbing 2 flights of stairs [29]. Electrocardiography and echocardiography were both normal. The participant consulted with his affiliated study clinician and completed benznidazole treatment.

Confirmed case-patient 2 was a 35-year-old man born in Guatemala who had resided in the US for >20 years. Occupational history included agricultural work, sometimes during the night. Recreational risk factors included backpacking and camping in Guatemala and the US, along with hunting and cleaning animals without glove protection [30–33]. The participant reported having seen the disease vector when shown insect photos, but had no known history of a triatomine bite and no knowledge about the disease. This patient had an unremarkable clinical history with neither reported comorbidities nor symptoms consistent with Chagas disease. He was unfortunately lost to follow-up after receiving confirmatory testing results (>3 outreach attempts were made by his physician). The patient did not undergo electrocardiography or echocardiography, nor was treatment administered.

**Figure 1.**
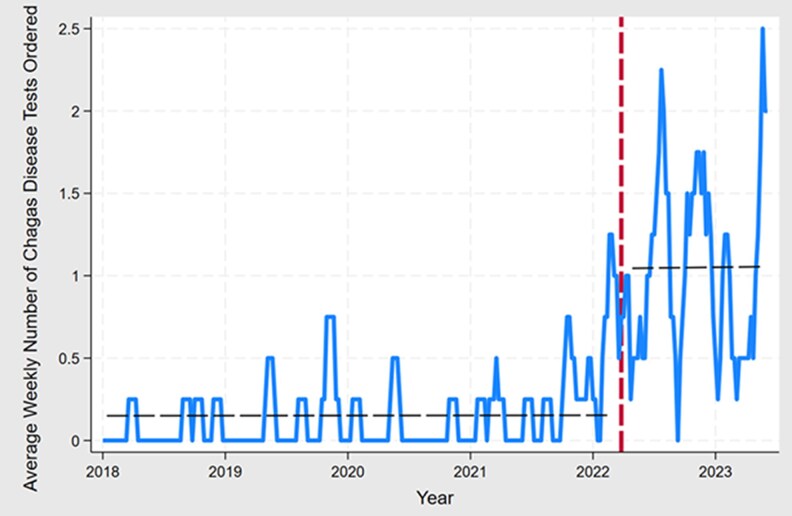
Time series analysis reveals a statistically significant upward trend in weekly Chagas disease diagnostic tests ordered post–provider educational campaign.

Figure 1 displays the average weekly number of Chagas disease diagnostic tests ordered by clinicians across the Prisma Health System in South Carolina. The red dashed line denotes the intervention (1 April 2022), and the horizontal dashed line marks the weekly average, pre- and postintervention. Visual inspection shows a noticeable increase in tests ordered during the postintervention period compared to the preintervention period. Summary *t*-test statistics show a statistically significant increase in the average number of weekly Chagas tests ordered during the preintervention period (mean ± standard deviation [SD], 0.16 ± 0.45 tests per week; range, 0–3) compared to the postintervention period (mean ± SD, 1.09 ± 0.92 tests per week; range, 0–4). An approximately 7-fold increase in the average weekly number of tests ordered was noted between the pre- and post-intervention period.

Supplementary Table 1 presents findings from several separate interrupted time series regression models. The main analysis appraised by a panel GEE model indicates a statistically significant (*P* = .002; 95% confidence interval [CI], .149–.665) weekly average increase in about 0.41 tests ordered (average marginal effect, 0.407). Sensitivity models confirmed this statistically significant increase in tests ordered during the postintervention period, with effect sizes ranging from 0.490 to 0.928 additional tests ordered per week. The logistic panel interrupted time series model noted a 32% increase (*P* = .003; 95% CI, .106–.525) in the chance that a Chagas test was ordered during the postintervention period (in comparison to the preintervention period). Overall, these results indicate a robust statistically significant increase in the intervention increasing clinician-ordered Chagas tests.

## DISCUSSION

Infectious diseases that disproportionally affect rural, low socioeconomic status, and/or immigrant populations are routinely underdiagnosed by clinicians due to lack of practitioner awareness, potential lack of perceived diagnostic urgency, healthcare access barriers, and a myriad of other disease-specific issues. This multicomponent clinical intervention demonstrated sustained impact, with *T cruzi* diagnostic orders significantly higher across the healthcare system for >2.5 years postintervention. The rate of provider-ordered *T cruzi* tests was 7-fold higher in the postintervention period. Chagas disease is a relatively rare condition many physicians have never heard of or never consider in diagnostic differentials [20, 34]; therefore, the ability to sustain routine diagnostic orders for several years is notable. The integration of Chagas disease “champions” into the diagnostic program along with demonstrated evidence of confirmed cases within the immediate patient population were key promoters of sustainability. The high seroprevalence, 2.9% of tested patients, further highlights the importance of this intervention. This high seroprevalence is comparable to studies performed in larger, Latin American–dominant US cities [15, 17], demonstrating that clinical education programs are warranted in moderate-size metropolitan and rural communities, especially as an estimated 6 million Latinx persons live in the rural US [35]. With the recent passage of clinical guidelines recommending that all human immunodeficiency virus–infected foreign-born Latin American residents be screened for *T cruzi* infection [36], which enabled federal financial support for diagnosis and treatment healthcare costs, the ability to effectively demonstrate evidence of veritable cases within additional healthcare systems across the US exists.

Though recent trends indicate a 23% increase in the Hispanic population since 2010, growth rates were highest in states without previously large Hispanic populations, many across the southeastern US [1]. Though trends show a slowing of Latin American immigration on the national level, there has been movement of Latin American–born persons within the US to more rural areas with affordable living conditions and job opportunities [37, 38]. This begs the need for enhanced education and awareness of rural providers, as several counties across South Carolina, North Carolina, Georgia, Alabama, Tennessee, and Florida have experienced at least a 50% increase in Hispanic populations in the last decade [1]. Several counties denoted in Supplementary Figure 1, particularly in Georgia, North Carolina, and Florida, have comparatively high rates of Latin American–born persons, and efforts to increase physician awareness and screening should be directed in these areas. Though Latin American–born persons are the highest-risk group, first-generation persons of Latin American descent with consistent cardiac symptoms should also be considered in screening programs due to the risk of congenital infection in the US. Additionally 43 000 cases exist among women of reproductive age, making screening of Latin American–born pregnant persons an important point for clinical management of cases within maternal-fetal medicine [13]. In order to provide high-quality, patient-focused care to foreign-born persons and to uncover the true burden of Chagas disease in the US, physician awareness campaigns should be implemented and screening enhanced on a national level.

Previous studies have shown that clinician disease awareness is low even among highest-risk groups such as Latin American–born persons [39]. Physician awareness campaigns and screening programs in the US have been few; however, they have been shown to be cost-effective [19, 21, 40]. Furthermore, these interventions have shown efficacy toward better physician knowledge of Chagas and implementation of testing [19, 21]. In this study, we saw a sustained 32% increase in physician ordered testing after the point of intervention. While education campaigns are a critical piece to addressing Chagas disease as a public health problem in the US, what set this intervention apart was the integration of paired “clinical Chagas champions” and demonstration of veritable patients within providers' practices. The champions program extended beyond providers to include all healthcare personnel involved in the clinical management spectrum, as clinic managers, medical billers, and laboratory personnel are important components to supporting the clinically complex treatment process and providing extra support for patients who are high risk of loss to follow-up. Additionally, the screening study not only demonstrated that a previously undiagnosed *T cruzi*–infected patient population did exist, but it also provided hands-on training experience for clinical personnel. Future clinical interventions, not just for *T cruzi*, should aim to incorporate these additional components to ensure long-term sustainability for timely detection and treatment of chronic, neglected infections, such as *Strongyloides stercoralis* or *Taenia solium* [41, 42].

Screening of individuals took place in 4 unique clinical settings across 5 South Carolina counties. Of note, Latin American–born individuals make up between 1% and 5% of the total population in counties where screening took place (Supplementary Figure 1). Clinics specializing in immigrant care and free health clinics were leveraged to bypass traditional financial and healthcare access barriers that these marginalized communities commonly incur [40]. The confirmed positive participants, along with 87% of the study population, were uninsured, and several participants cited barriers to seeking healthcare. Other barriers that prevent these highest-risk groups of foreign-born persons from seeking care include difficulty navigating the US healthcare system, social stigma, and language and transportation constraints; these issues are exacerbated in moderate-size cities or rural areas, which lack these built environment support systems [14, 43]. Lack of public knowledge of Chagas disease is a further challenge, as high-risk persons may be unaware of their risk for infection. In this study, nearly 75% of participants had not previously heard of the infection, which decreases their empowerment to seek out screening during unrelated healthcare encounters (eg, vaccinations or dental exams). Collectively, these barriers and challenges should be considered when prospective Chagas disease screening programs are implemented to ensure that vulnerable patients are provided with quality health care for this neglected parasitic infection.

Healthcare personnel and clinicians face various barriers to conducting *T cruzi* screening, including low awareness or outdated infection knowledge, lack of language services at smaller clinics, and low treatment efficacy among adult, indeterminant cases [14]. Further, well-known challenges exist with diagnostic screening including low positive predictive value of US Food and Drug Administration–approved testing platforms and heterogeneity in test results due to variability in circulating discrete typing unit by originating country [17, 44]. Clinicians have also cited challenges with time constraints, as additional time is needed to invest in screening programs for confirmatory testing, referrals, and follow-up appointments on top of current caseloads [19]. Other barriers faced by clinicians screening for Chagas disease include high loss to follow-up or medication discontinuation rates, as previous studies have shown that up to 80% of *T cruzi–*positive patients discontinue clinical care before treatment can be initiated [40, 45]. Our study exemplified these same issues in smaller, nonmetropolitan areas. Loss to follow-up was an issue within our study patient population, as one-third of screened positive patients failed to return for confirmation testing and only 1 of 2 confirmed patients received antiparasitic benznidazole treatment. Concern for political persecution due to immigration status could have been a motivating factor in these patients' lack of follow-up [46]; however, this work was done with healthcare experts trained in cultural sensitivity and experience working with this population in an effort to boost clinical management. This study's issues are not unique, as <0.3% of those with Chagas in the US have been treated, despite CDC guidelines strongly recommending screening and treatment for persons <50 years of age with Chagas disease [47].

A few study limitations are worth noting. First, a survey of physician knowledge pre- and postintervention was not performed, as the investigator team felt that count of diagnostic tests was a more meaningful measure of impact. However, this limits the potential sensitivity of this study, and future interventions like this should include provider knowledge, attitudes, and practices surveys as proxy measures. The *T cruzi* screening component was limited by a convenience sampling of Latin American–born individuals presenting for clinical or dental care. Therefore, selection bias could be present, as those not experiencing symptoms or health issues may have been less likely to present for care and thus be recruited. Third, the quasi-experimental design limited our ability to control other potential factors leading to increased testing. In an ideal setting, a randomized controlled trial of providers receiving the intervention versus a control group would yield the optimal rigorous determination of the intervention's true impact. Last, many participants in this study were uninsured; however, established infrastructure within this hospital system facilitated our participants' ability to receive care. Therefore, the sustainability of Chagas screening programs across other healthcare systems will depend on health services mechanisms that ensure access to care through community health insurance programs or other established protocols.

## CONCLUSIONS

Chagas disease screening is inadequate in the US, and barriers including physician awareness and healthcare access leave >99% of veritable cases undiagnosed and untreated. With Latin American–born population movement to more rural and atypical metropolitan areas across the US, awareness and testing outside of the areas with historically high Latin American–born populations should be increased. The 2.9% seroprevalence found in this study highlights the fact that disease cases exist in relatively high numbers in moderate-size cities and rural communities. This study demonstrated that sustainable *T cruzi* testing requires more than a 1-time educational talk—integrating job-specific knowledge across the clinical workforce and providing guided case examples can improve the long-term sustainability of provider testing. As healthcare barriers for vulnerable populations may increase in the foreseeable future due to recent federal changes, the need for programs like that described in this study will also grow. Timely identification of chronic, insidious parasitic infections is not only critical for the patient but also provides cost-savings to the healthcare system at large.

## Supplementary Material

ofaf467_Supplementary_Data

## Supplementary Data

Supplementary materials are available at *Open Forum Infectious Diseases* online. Consisting of data provided by the authors to benefit the reader, the posted materials are not copyedited and are the sole responsibility of the authors, so questions or comments should be addressed to the corresponding author.
