# Supplementary material for: An Evidence-Based Intervention to Increase Trypanosoma cruzi, a Neglected Parasitic Infection, Diagnosis in Rural and Moderate-Size-City US Clinics
Source: Open Forum Infect Dis. 2025 Aug 14;12(8):ofaf467. doi: 10.1093/ofid/ofaf467 (PMC12372667; doi:10.1093/ofid/ofaf467)
Supplement: ofaf467_Supplementary_Data [file ofaf467_supplementary_data.zip › Supplemental Table 1.docx]

Supplemental Table 1. Additional interrupted time series models were executed to demonstrate consistency of findings across various statistical approaches. These models assessed the impact of the Prisma Health System Intervention on Chagas disease tests ordered by clinicians.

|  | AME (S.E.) | P | 95% CIs |
| --- | --- | --- | --- |
| Generalized Estimating Equations Model |  |  |  |
| Intervention Effect | 0.407 (0.132) | 0.002 | 0.149 – 0.665 |
| Linear Trend | 0.001 (0.001) | 0.115 | -0.000 – 0.003 |
| Pooled Interrupted Time Series Model |  |  |  |
| Intervention Effect | 0.490 (0.187) | 0.009 | 0.123 – 0.856 |
| Linear trend | 0.001 (0.001) | 0.076 | -0.000 – 0.003 |
| Panel Poisson Model | |  |  |
| Intervention effect | 0.928 (0.324) | 0.004 | -.293 – 0.1564 |
| Linear trend | 0.007 (0.002) | 0.001 | 0.003 – 0.011 |
| Panel Negative Binomial Model |  |  |  |
| Intervention Effect | 0.928 (0.324) | 0.004 | 0.293 – 0.1564 |
| Linear Trend | 0.007 (0.002) | 0.001 | 0.003 – 0.011 |
| Panel Logistic Model |  |  |  |
| Intervention Effect | 0.321 (0.110) | 0.003 | 0.106 – 0.536 |
| Linear Trend | 0.001 (0.000) | 0.002 | 0.000 – 0.002 |
| *Notes: AME = average marginal effects, S.E. = standard errors, P = p-value, CIs = 95% confidence intervals. Fifth-order lag, month, and quadratic trend are not presented to conserve space within the table.* | | | |
