# Supplementary figures and images for: An Evidence-Based Intervention to Increase Trypanosoma cruzi, a Neglected Parasitic Infection, Diagnosis in Rural and Moderate-Size-City US Clinics
Source: Open Forum Infect Dis. 2025 Aug 14;12(8):ofaf467. doi: 10.1093/ofid/ofaf467 (PMC12372667; doi:10.1093/ofid/ofaf467)

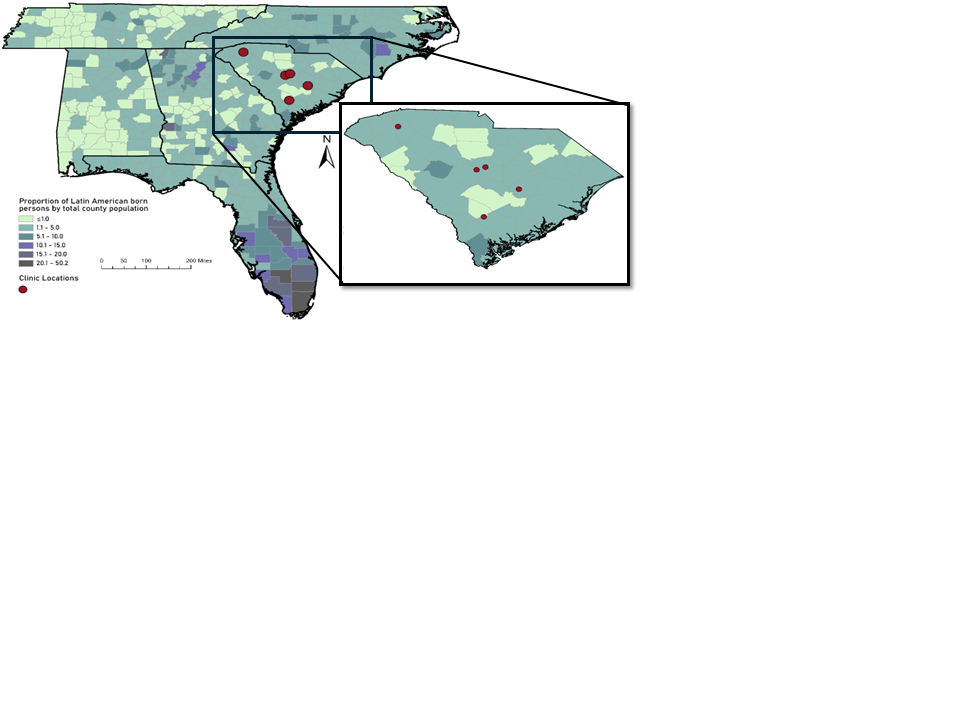

Supplement: ofaf467_Supplementary_Data [file ofaf467_supplementary_data.zip › Supplemental Figure 1.tif]
